# Proteomics analysis of the peritoneal dialysate effluent reveals the presence of calcium-regulation proteins and acute inflammatory response

**DOI:** 10.1186/1559-0275-11-17

**Published:** 2014-04-17

**Authors:** Elisabete Oliveira, José E Araújo, Silvana Gómez-Meire, Carlos Lodeiro, Cristina Perez-Melon, Elena Iglesias-Lamas, Alfonso Otero-Glez, José L Capelo, Hugo M Santos

**Affiliations:** 1 BIOSCOPE Research Group. REQUIMTE, Departamento de Química. Faculdade de Ciências e Tecnologia, Universidade NOVA de Lisboa, Caparica, 2829-516, Portugal; 2 PROTEOMASS Scientific Society, Madan Parque, Rua dos Inventores, Caparica, 2825-182, Portugal; 3 SING Group. Informatics Department. Higher Technical School of Computer Engineering, University of Vigo, Ourense, Spain; 4 Servicio de Nefrología, Complejo Hospitalario Universitario de Ourense, Ourense, 32004, España

**Keywords:** Peritoneal dialysis effluent, 2D-Gel Electrophoresis, Protein identification, Proteomics

## Abstract

**Background:**

Peritoneal dialysis (PD) is a form of renal replacement used for advanced chronic kidney disease. PD effluent holds a great potential for biomarker discovery for diagnosis and prognosis. In this study a novel approach to unravelling the proteome of PD effluent based-on dithiothreitol depletion followed by 2D-SDS-PAGE and protein identification using tandem mass spectrometry is proposed.

**Results:**

A total of 49 spots were analysed revealing 25 proteins differentially expressed, among them many proteins involved in calcium regulation.

**Conclusions:**

Remarkably, a group of proteins dealing with calcium metabolism and calcium regulation has been found to be lost through peritoneal dialysate effluent, giving thus a potential explanation to the calcification of soft tissues in patients subjected to peritoneal dialysis and kidney injury. Comparison of literature dealing with PD is difficult due to differences in sample treatment and analytical methodologies.

## Background

Proteomics is a powerful technology with high-throughput capabilities in evaluating complex protein mixtures from biological samples [1]. Through proteomics it has been possible to establish that certain diseases can be traced from alterations of certain proteins in plasma [2]. In addition to plasma, there are other biological fluids that contain proteins and that hold great potential to be used as indicative of disease, such as cerebrospinal fluid, pleural, pericardial effusions, urine, and peritoneal dialysate [3-7]. For example, urine has been used for studies involving kidney and bladder diseases [3,4], whilst peritoneal dialysate has been used in endometriosis [1], and ovarian cancer [6,7] studies.

The increasing in plasma levels of endogenous or exogenous toxins is often associated with diseases [8]. When there is renal failure, removal of these toxins from blood circulation can be done by effective methods, as extracorporeal blood purification and peritoneal dialysis (PD). Both methods are mainly used as renal replacement therapy, as artificial kidney, in patients with end-stage renal disease and acute kidney injury [9-12].

PD produces a fluid known as peritoneal dialysate effluent (PDE). PDE can be used for clinical diagnosis as it contains complement factors, hormones, acute phase proteins, coagulation factors and apolypoproteins [13-17]. The analysis of PDE by proteomics is relatively recent. As for the analysis of any complex proteome, the results in terms of proteins identified is limited by the presence of the most abundant proteins (MAPs), and for the strategy followed to circumvent this drawback. The first problem deals with the fact that the concentration of proteins in complex proteomes can vary over 10 orders of magnitude whilst the analytical technologies currently available have 2 to 4 orders of magnitude in dynamic range for protein detection [2]. Therefore, not all proteins can be determined only on base on the analytical technologies available in the laboratory. Different solutions have been developed to avoid this problem, relaying in the separation of the MAPs, or in the equalization of protein concentration of the proteins present in the PDE [18,19]. The aim is to reduce the concentration of MAPs at levels in which they do not interfere with the detection of other low-abundance proteins. Recently, DL-Dithiotreitol (DTT) has been reported as a cheap and easy way to deplete high abundance proteins to simplify the protein content in human serum [20,21]. Whichever the solution adopted, this manuscript shows that the data reported to date dealing with the proteome of PDE is somewhat inconsistent, as from five works compared only one protein was found redundant, this is, the majority of the proteins of interest identified are not consistently reported. Both, the type of patients selected as well as the differences in the analytical procedures might be greatly influencing the results. In the present work DTT has been used to partially deplete peritoneal dialysate effluent with success. A revision of the proteins found to date in literature dealing with PDE is also presented and compared with the ones found in this work. A total of 49 proteins have been identified resulting in 25 non-redundant proteins.

## Results and discussion

### Analysis of individual peritoneal dialysate effluent samples using 2-DE

**Figure 1:**
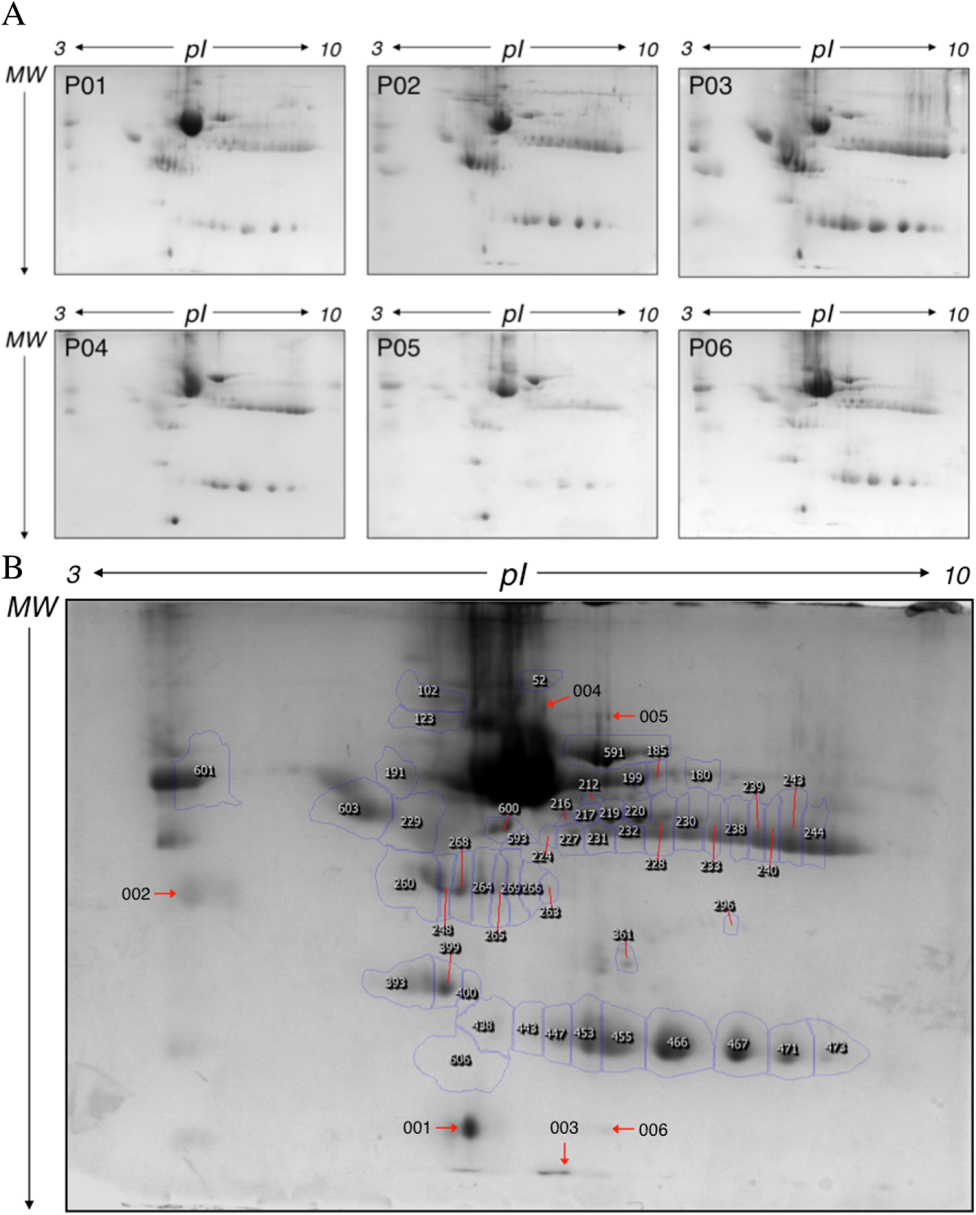
**2D-GE gels of peritoneal dialysis effluent. (A)** Representative 2D-GE gels of peritoneal dialysis effluent of each patient used in the study. **(B)** Reference gel with spot annotation. Fist dimension was performed in IPG strips pH 3–10, 7 cm and second dimension was done in SDS-PAGE 12% acrylamide/bis-acrylamide 37.5:1.

In the present study, samples of peritoneal dialysate effluent were collect from six patients from the *Complexo Hospitalario Universitario de Ourense (CHUO)*. The clinical data of these patients is shown in Table 1. Before analysis the six samples of peritoneal fluid were depleted with DTT as described in Section 2.4 and quantified by Bradford assay (total protein content: P01 4.8 ± 0.3 μg/μL; P02 5.1 ± 0.2 μg/μL; P03 6.3 ± 0.2 μg/μL; P04 5.3 ± 0.1 μg/μL; P05 4.9 ± 0.2 μg/μL; P06 5.7 ± 0.1 μg/μL; see Figure 1 of Additional file 1: Figure S1SM, for further details). DTT is usually applied to disrupt intramolecular and intermolecular disulphide bonds, helping to unfold proteins rich in such bonds, leading to their precipitation. As the majority of the most abundant proteins, such as albumin and serotransferin, are reach in disulphide bonds, they are preferentially depleted, although not totally eliminated. Thus, the visualization of other minor proteins is allowed whilst major proteins are not totally precipitated [20,21].

**Table 1:** Clinical information about the patients tested on this study

	**Age**	**Gender**	**Classification**	**Total time in PD (days)**	**Kt/V (s)**^**a**^	**PET**^**b**^	**Alb**	**Ca**	**P**	**PTH**	**Vit. D**
							**(μg/mL)**	**(μg/mL)**	**(μg/mL)**	**(pg/mL)**^**c**^	**(ng/mL)**
P01	69	M	Unknown	96	1.93	0.84	31	98	61	233	5.09
P02	80	F	Ischemic	45	2.3	0.62	30	94	57	237	5.4
P03	88	F	Unknown	72	1.5	0.76	21	91	30	49	<3
P04	73	M	Diabetes	Unknown	1.9	-	28	110	26	234	<4
P05	54	M	Polycystic	34	2.1	0.75	33	85	59	636	82
P06	51	M	Unknown	38	3	0.82	30	78	49	374	7.79

^a^K dialyzer clearance of urea, t is the dialysis time, V is the volume of distribution of urea; ^b^Peritoneal equilibrium test; ^c^Parathyroid hormone.

**Figure 2:**
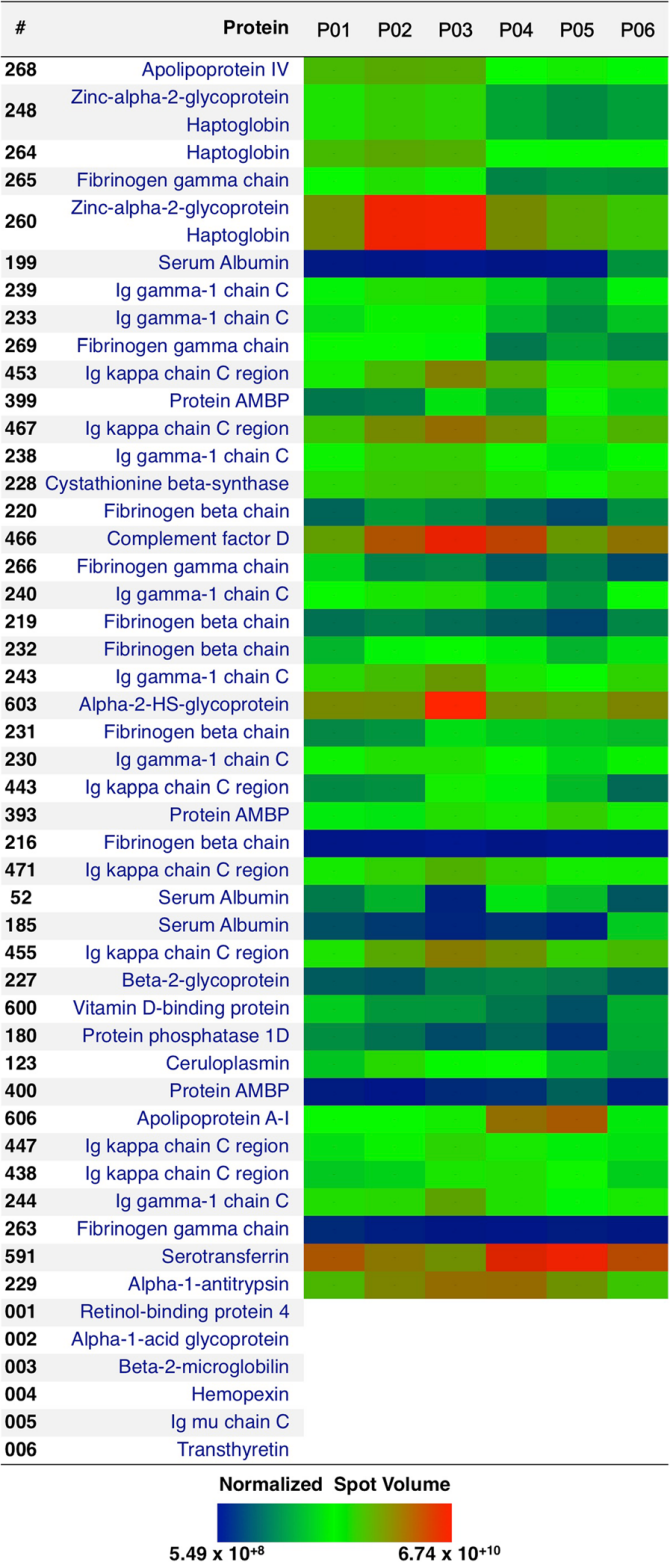
Comparative level of protein abundance in peritoneal dialysate effluent.

In order to analyze the differences among the patients, 2D gels were carried out by triplicate for each patient. A total of 100 μg of protein was loaded onto pH 3–10 strips, and then proteins were visualized with CBB. A representative gel is presented in Figure 1. The 2D gels, were analysed using Progenesis SameSpots software as described in Section 2.9, and 49 spots were detected and analysed (Figure 1B). Gel spots were excised and subjected to in-gel digestion and MALDI-TOF/TOF analysis. Detailed information on the identified proteins is given in Additional file 2: Table S1SM and Additional file 3: Table S2SM. A total of 49 spots were excised and analysed, rendering 49 identifications, after removing redundancy only 25 different proteins were identified, see Figure 2. Some proteins, such as Ig kappa chain C region as well as Fibrinogen beta chain were found in as much as 7 and 9 spots, respectively. This redundancy can be linked with the existence of post- translational modifications (PTMs) or the presence of proteases in the PDE, which might be responsible for the proteolysis of the proteins, rendering this way a solution rich in protein fragments. These two effects would lead to identify the same protein in different spots. Therefore, and for future works, it is recommended to collect the PDE with a cocktail of protease inhibitors as well as the analysis of PTMs.

### The proteins identified, an integrative analysis

**Figure 3:**
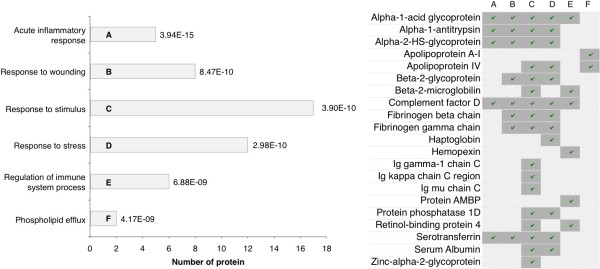
**Gene ontology annotation, the horizontal bars represent the number of protein identified in peritoneal dialysis effluent and involved in each biological process.** The p-value calculated for each biological pathway is shown over the bar. **A** acute inflammatory response; **B** response to wounding; **C** response to stimulus; **D** response to stress; **E** response of immune system process; **F** phospholipid efflux. In the table on the right are shown the protein involved in each biological pathway.

An integrate analysis of all proteins identified was performed with Cytoscape (v2.8.3). The protein-protein interaction network was imported from Intact ( http://www.ebi.ac.uk/intact/) [22] and contains 318 proteins and 340 protein-protein interactions (data not shown). To identify the relevant biological pathways that were altered, BiMGO [23] was used to find gene ontology (GO) classes of biological processes that were enriched among the identified proteins. Thus, the most significant biological pathways found were, (i) acute inflammatory response, (ii) response to wounding, (iii) response to stress, (iv) response to stimulus, (v) regulation of immune system processes and (vi) phospholipid efflux (see Figure 3). Comments on some of the most relevant proteins identified are given below.

Fetuin-A forms soluble complexes with calcium and phosphate that otherwise would not be soluble in blood as well as participates in inflammatory response [24-26]. Furthermore, this protein has been identified as a potent inhibitor of pathological calcification, as mice deficient in Fetuin-A suffers of systematic calcification of soft tissues [27,28]. In addition to this finding, protein AMBP, also found, inhibits calcium oxalate crystallization. Both, Fetuin-A and protein AMBP are involved in calcium regulation. Further to he above proteins it was also found vitamin-D-binding protein. This proteins is also involved in the metabolism of calcium. Calcification of soft tissues is a problem in end-terminal patients with renal failure. As those proteins above mentioned were found in all PDEs studied in this work, the loss of such proteins through the peritoneum, might potentially explain the calcification of soft tissues in patients subjected to PD. This is such a serious and important issue that it deserves to be dealt with in future research.

The Ig kappa protein found is an antibody, which presents two types of light chains in humans, the kappa chain and the lambda chain. Both types are highly correlated in humans, varying the normal ratio of kappa to lambda from 0.26 to 1.65. High levels of these proteins are correlated with kidney diseases [29]. This protein has been also linked to inflammatory processes in the human body. Therefore, its presence in PDE was not unexpected. This protein could be used as a potential biomarker of disease progression as its concentration could be potentially linked with the status of the patient. Also, the kappa/lambda ratios might deliver information for diagnostic purposes, especially if the ratio found in the PD is linked with the one found in serum.

Retinol-binding protein 4, RET4, and transthyretin (TTHY) are close correlated. RET4 is the specific carrier for vitamin A (retinol) in the blood. The RET-VitA complex interacts with TTHY, being thus prevented the loss of the complex through the kidney glomeruli. The presence of both proteins in the PDE could indicate a failure in the formation of the RET-VitA complex in patients with kidney injury or the loss of the complex trough the peritoneum, and therefore an explanation to problems related with levels of vitamin A in the body of such type of patients is advanced. Also, it would be desirable to analyse the content of the vitamin A in both serum and PDE.

Alpha-1-microglobulin (A1M) is a protein that acts suppressing the heme-group, and it is believed to protect cell and tissues against the damage induced by high concentrations of free hemoglobin in blood [30].

The presence of fibrinogen (alfa and beta chains) may suggest an excessive loss of this protein through the peritoneal liquid, which may lead to disturbed function of fibrinogen and also to bleeding or thromboembolic complications [28,29].

Alpha 1-antitrypsin, A1AT acts as an inhibitor of proteases, protecting tissues from enzymes of inflammatory cells. Its deficiency can cause complications such as emphysema, or chronic obstructive pulmonary disease in adults and cirrhosis in adults or children [31,32].

### Comparison of literature dealing with PDE

**Figure 4:**
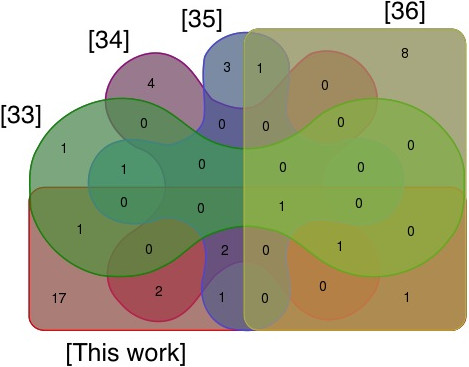
**Venn diagrams - Groups of proteins per article and mutual proteins between them.** (Sritippayawan et al*.*, [33]); (Tyan et al., [34]); (Wang et al., [35]); (Wang et al., [36]); and the proteins identified in this work.

The literature available about PDE to date is scarce, and yet some conclusions can be drawn in advance. First, the studies developed to date have focused in different types of PD patients, making difficult a comparison. There is an urgent need for more oriented studies, i.e. focusing on one disease. Ideally, such studies should be done by different research teams and in different countries. Eventually, this would help to unravel if progress of renal failure can be monitored from the protein content of the PDE. Second, there is also a problem related to the handling and subsequent sample treatment of the PDE. This problem is highlighted in Figure 4, where different studies dealing with PDE are compared. As may be seen, although 2D-GE was used as the main analytical tool in all the works compared, the number of proteins found in common between them is almost negligible. It is not clear if this happens because of the differences in the type of patients assessed or in the sample treatment used in each work or because of both. Most likely, both differences influence the result.

## Conclusions

Through this work it has been demonstrated that the proteome of the peritoneal dialysis effluent is far from being well established. A total of 25 proteins differentially expressed have been identified among 6 patients with PD. It is concluded that studies dealing with PDE are difficult to compare if the methodology used to treat the samples and to identify the proteins are not the same. It has been shown that the DTT-based depletion method is a cheap alternative to be considered to other expensive approaches. The most remarkable result found deals with the lost of proteins trough the PDE related with the metabolism as well as regulation of calcium in the human body. This finding might be directly linked to the calcification of soft tissues, in patients subjected to PD, and opens new insights into the potential use of PDE as a sample for diagnosis and prognosis of patients with renal failure.

## Methods

### Chemicals and reagents

All reagents used were HPLC grade or electrophoresis grade. Albumin, from bovine serum (BSA), urea, thiourea, 3-[(3-Cholamidopropyl) dimethylammonio]-1- propanesulfonate (CHAPS), β-mercaptoethanol, glycerol 86-88%, bradford reagent, coomassie blue R-250 (CBB), DL-dithiotreitol (DTT), iodoacetamide (IAA), trypsin sequencing trifluoroacetic acid (TFA), sodium deoxycholate (DOC) and acrylamide/bis-acrylamide 30% solution (37.5:1) were purchased from Sigma-Aldrich (St. Louis, USA). (N, N, N’, N’–tetramethylethylene-diamine (TMED), ammonium persulphate (APS), glycine were purchased from NZYTech (Lisbon, Portugal). Ampholytes pH 3–10, formic acid, and ammonium bicarbonate were purchased from Fluka (Steinheim, Germany). Hydrochloride acid (HCl), glacial acetic acid, tris-base, trichloroacetic acid (TCA), sodium dodecyl sulfate (SDS), methanol, acetonitrile were purchased from Panreac (Barcelona, Spain). Bromophenol blue was from Riedel-de Haën. Agarose and Mineral oil were purchased from Biorad (Hercules, USA).

Molecular weight marker for gel electrophoresis NZYColour Protein Marker II was purchased from NZYTech (Lisbon, Portugal). α-Cyano-4-hydroxycinnamic acid puriss for MALDI-MS from Fluka was used as MALDI matrix.

### Samples

Peritoneal dialysate (PD) liquid was collected from six anonymous patients from the Hospital of Ourense, Spain. Once taken, the samples were aliquoted in the laboratory and stored at -80°C until further use. The study was approved by the ethics committee of Galicia (Spain). The clinical data of these patients is shown in Table 1.

### Apparatus

Protean IEF cell, IPG strips pH 3–10 (7 cm), IEF focusing tray with lid, SDS-PAGE (SDS-Polyacrylamide gel) electrophoresis cell (model Mini-PROTEAN 3) were from Bio-Rad, following the manufacturer’s instructions. The image of the gel after staining was acquired in a PROPIC II DigiLab Genomic Solutions USA. Protein digestion was done in safe-lock tubes of 1.5 mL from Eppendorf (Hamburg, Germany). A vacuum concentrator centrifuge model UNIVAPO 150 ECH SpeedVac and a vacuum pump model UNIJET II were used for sample drying and sample pre-concentration. A mini incubator from Labnet was used for gel washing, for protein reduction and for protein alkylation steps. The centrifuge MPW-350 and MPW-65R were from MPW Med. Instruments. Absorption spectra of samples were recorded as microliter samples using a NANODROP ND-1000 Spectrophotometer from Thermo Scientific (USA). Protein identification was done in an Ultraflex II MALDI-TOF/TOF instrument from Bruker Daltonics.

### Protein depletion

All samples were precipitated in triplicate in order to access reproducibility of the protocol. Peritoneal dialysate aliquots of 750 μL were made up to 50 mM DTT. Samples were incubated until precipitate was observed. The supernatants were harvested by centrifugation at 14.000×g for 20 minutes at 4°C. The proteins present on the resulting supernatant were precipitated by DOC/TCA. Briefly samples were incubated with 0.02% (v/v) DOC for 30 minutes on ice and then TCA 100% (w/v) were added to a final concentration of 15% (v/v). Then samples were incubated 2 hours on ice. The protein pellets were harvested by centrifugation at 14.000×g for 20 minutes at 4°C. Then pellets were washed 3 times with 200 μL of cooled acetone to remove TCA. Protein pellets were stored at -80°C for further processing.

### 2D gel electrophoresis

The protein pellets obtained after precipitation were solubilized in 200 μL of lysis buffer (7 M urea, 2 M thiourea, 30 mM Tris, 2% (w/v) CHAPS, 0.2% (v/v) ampholytes pH 3–10 and 50 mM DTT). Samples were sonicated using a 1 mm diameter probe for 6x10 seconds on ice at 50% sonication amplitude. Insoluble mater was removed by centrifugation (20 minutes 14.000×g at 4°C). Protein concentration was determined using a Bradford assay. Samples containing about 150 μg of protein from each patient were mixed with rehydration buffer (7 M urea, 2 M thiourea, 2% (w/v) CHAPS, 0.2% (v/v) ampholytes pH 3–10, 50 mM DTT and traces of bromophenol blue) to obtain a protein solution of 0.8 μg/μL. Sample loading on IPG-Strip was carried out following the rehydration loading method. Briefly, 125 μL of the rehydration buffer containing 0.8 μg/μL of total protein was slowly delivered as a stipe of liquid on a strip holder. Then, the protective cover foil from the IPG strip was removed and placed gel facing down over the strip of rehydration buffer containing protein. Then it was covered with 1 mL of mineral oil. IPG strips were allowed to rehydrate overnight at 20°C. After rehydration/sample loading gel strips were focused. The strips were removed from the rehydration tray and the oil was drained, and then transferred to the focusing tray. The focusing tray was placed into the PROTEAN IEF Cell. The focusing was performed at voltage: 4000 Volts, time: 6 h30 minutes, maximum current: 50 μA/gel, Volt-Hours: 10.000 V-hr. When the electrophoresis run finished, the IPG strips were removed and were incubated with equilibration buffer (6 M urea, 75 mM Tris pH 8.8, 20% glycerol (v/v), 2% (w/v) SDS, traces of bromophenol blue) as follows 15 minutes incubation with 2.5 mL of equilibration buffer containing 2% (w/v) of DTT, followed by 15 minutes incubation with 2.5 mL of equilibration buffer containing 2.5% (w/v) of IAA. The IPG strips were removed from the equilibration tray and clip briefly into the graduated cylinder containing the running buffer. The strip gel was placed side up and onto the back plate of the SDS-PAGE gel. The IPG well of the gel was overlay with agarose sealing solution (0.5% w/v prepared with 50 mL of Lammeli running buffer and traces of bromophenol blue). After agarose solidification, the electrophoresis was conducted at 200 V (constant voltage), 400 mA, 50 minutes.

### Gel staining and image analysis

Finished the gel electrophoresis, the gel was fixed for 30 minutes with 40% (v/v) ethanol and 10% (v/v) acetic acid and then stained overnight with coomassie brilliant blue. Gels were distained with 40% (v/v) methanol, 10% (v/v) acetic acid until a clear background was observed. Gel imaging was carried out with a ProPicII-robot using 14 ms of exposure time and a resolution of 70 μm. Gel piking were done with the same equipment. Progenesis SameSpots software (v 4.0, NonLinear Dynamics) was used for gel alignment, spot detection, spot quantification, and normalisation for total spot volume in each gel. Data were statistically compared using the incorporated statistical package. Significant between-patients differences for each protein were calculated using Student's t test, considering a p value of < 0.05 as statistically significant and a volume ratio > 1.5 (increase or decrease).

### In-gel protein digestion

After spot piking the spots were transferred to 0.5-mL Lo-bind tubes. Gel spots were washed with water and then with 50% acetonitrile/25 mM Ambic until the blue color disappears. For protein reduction gel spots were incubated for 60 min with 10 mM dithiothreitol in 25 mM Ambic at 37°C followed by alkylation at room temperature in the drack with 55 mM iodoacetamide in 25 mM Ambic. Prior to trypsin digestion, gel spots were washed with 25 mM Ambic and dehydrated with acetonitrile. Then, 15 μL of trypsin (0.02 μg/μL in Ambic 12.5 mM/9% acetonitrile) was added to the gel spots and incubated for 60 min on ice. After this time, gel spots were inspected and all the trypsin solution not absorbed into the gel were removed and the gels were covered with 25–50 μL of 12.5 mM Ambic depending on the spot volume. The samples were incubated 12 h at 37°C. Then 25 μL formic acid 5% (v/v) was added to quench enzymatic activity. The supernatant was transferred to new lo-bind tube and the peptides were further extracted from the gel with 50% acetonitrile/5% formic acid. Samples were dried-down and stored at -20°C until MS analysis.

### MALDI-TOF-MS/MS analysis

Prior to analysis, samples were ressuspended in 10 μL of formic acid 0.3% and 1 μL of sample was hand-spotted onto a MALDI target plate (384-spot ground steel plate) then 1 μL of a 7 mg/mL solution of a-cyano-4-hydroxycinnamic acid matrix in 0.1% (v/v) TFA and 50% (v/v) ACN was added and allowed to air dry. The mass spectrometer was operated in positive ion mode using a reflectron, and thus, spectra were acquired in the m/z range of 600–3500. A total of 500 spectra were acquired for each sample at a laser frequency of 50 Hz. External calibration was preformed with the [M + H] + monoisotopic peaks of bradykinin 1–7 (m/z 757.3992), angiotensin II (m/z 1046.5418), angiotensin I (m/z 1296.6848) substance P (m/z 1758.9326), ACTH clip 1–17 (m/z 2093.0862), ACTH18–39 (m/z 2465.1983) and somatostatin 28 (m/z 3147.4710). Peptide mass fingerprints (PMF) were searched with MASCOT search engine with the following parameters: (i) SwissProt Database2012_04 (535698 sequences; 190107059 residues); (ii) molecular weight of protein: all; (iii) one missed cleavage; (iv) fixed modifications: carbamidomethylation (C); (v) variable modifications: oxidation of methionine and (vi) peptide tolerance up to 50 ppm after close-external calibration. The significance threshold was set to a minimum of 95% (p ≤ 0.05). A match was considered successful when protein identification score is located out of the random region and the protein analysed scores first.

## Abbreviations

A1AT: Alpha 1-antitrypsin; A1M: Alpha-1-microglobulin; APS: Ammonium persulphate; BSA: Albumin from bovine serum; CBB: Coomassie blue R-250; CHAPS: 3-[(3-Cholamidopropyl) dimethylammonio]-1- propanesulfonate; DOC: Sodium deoxycholate; DTT: DL-Dithiotreitol; IAA: Iodoacetamide; MAPs: Most abundant proteins; PD: Peritoneal dialysis; PDE: Peritoneal dialysate effluent; PTMs: Post- translational modifications; SDS: Sodium dodecyl sulfate; TCA: Trichloroacetic acid; TFA: Trifluoroacetic acid; TMED: N, N, N’, N’–tetramethylethylene-diamine; TTHY: Transthyretin.

## Competing interest

The authors declare that they have no competing interests.

## Authors’ contributions

AOG, JLC, and HMS participated in the conception and study design. CPM and EIL collected the samples. EO, JEA and HMS carried out the 2D-GE and mass spectrometry analysis of the samples. JLC and HMS wrote the manuscript. SGM was involved in data analysis and interpretation. SGM, AOG, CPM, CL and EIL revised the manuscript and added valuable suggestions for improving it. CL, JLC and AOG provided financial Support. All authors read and approved the final manuscript.

## Supplementary Material

Additional file 1: Figure S1SM Protein concentration in the peritoneal dialysate effluents used in the study. Protein concentration was determined by Bradford assay. Click here for file

Additional file 2: Table S1SM Proteins identified in 2D-gels of peritoneal dialysis effluent. Click here for file

Additional file 3: Table S2SM Proteins identified in 2D-gels of peritoneal dialysis effluent: Mascot Score, protein sequence coverage for the identified proteins. Click here for file
