# Supplementary material for: Proteomics analysis of the peritoneal dialysate effluent reveals the presence of calcium-regulation proteins and acute inflammatory response
Source: Clin Proteomics. 2014 Apr 17;11(1):17. doi: 10.1186/1559-0275-11-17 (PMC4022211; doi:10.1186/1559-0275-11-17)

**Additional file 1: figure S1SM**

Protein concentration in the peritoneal dialysate effluents used in the study. Protein concentration was determined by Bradford assay.


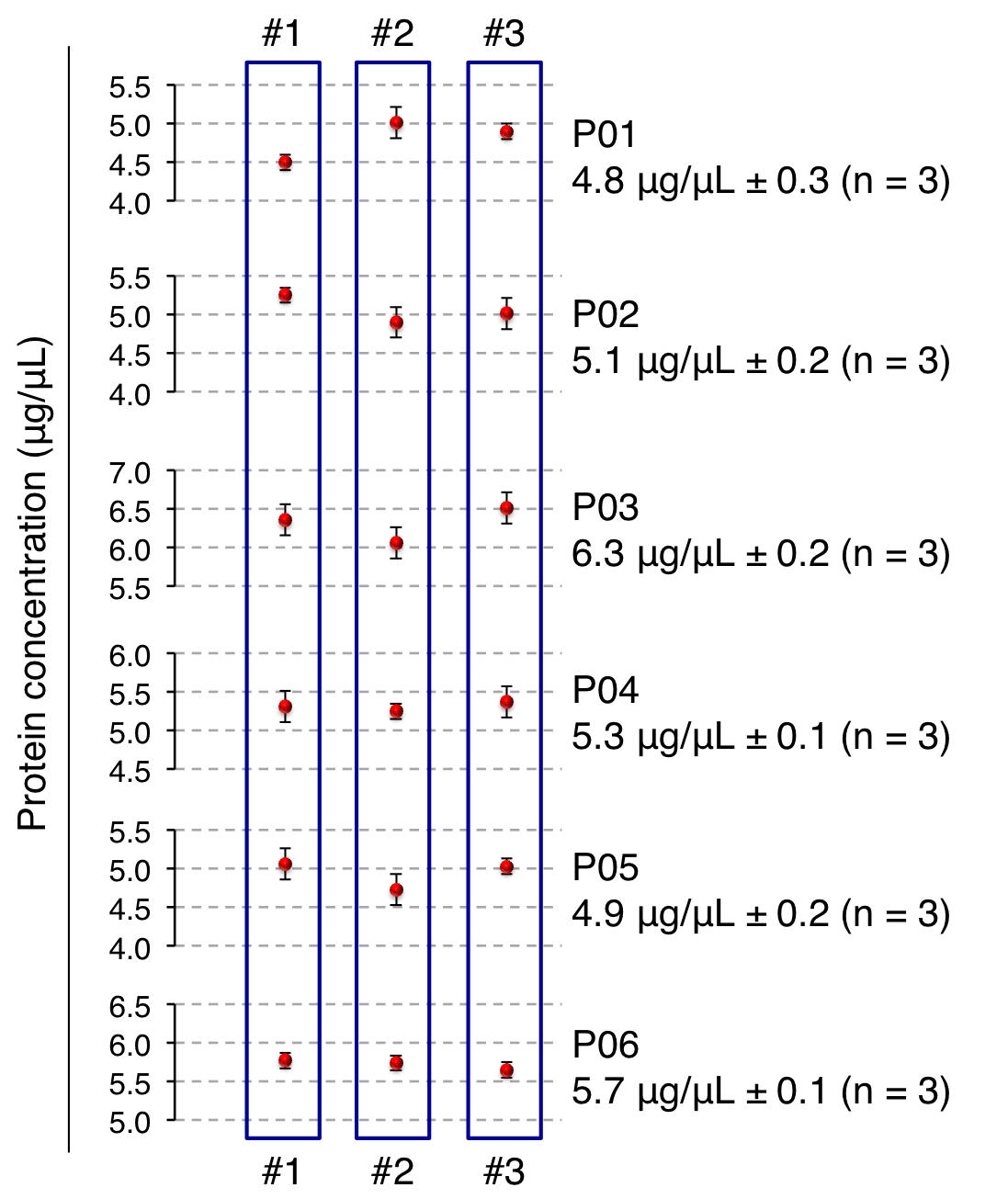

Supplement: Additional file 1: Figure S1SM — Protein concentration in the peritoneal dialysate effluents used in the study. Protein concentration was determined by Bradford assay. [file 1559-0275-11-17-S1.doc]
