# Supplementary material for: Proteomics analysis of the peritoneal dialysate effluent reveals the presence of calcium-regulation proteins and acute inflammatory response
Source: Clin Proteomics. 2014 Apr 17;11(1):17. doi: 10.1186/1559-0275-11-17 (PMC4022211; doi:10.1186/1559-0275-11-17)
Supplement: Additional file 2: Table S1SM — Proteins identified in 2D-gels of peritoneal dialysis effluent. [file 1559-0275-11-17-S2.doc]

**Additional file 2:**

**Table S1SM.** Proteins identified in 2D-gels of peritoneal dialysis effluent.

| **Spot Nr.** | **Anova (p)** | **Fold** | **Protein** | **Protein Description** | **Protein** | **Protein** | **Average Normalized Volumes** | | | | | |
| --- | --- | --- | --- | --- | --- | --- | --- | --- | --- | --- | --- | --- |
|  |  |  | **Accession** |  | **pI** | **MW** | **P01** | **P02** | **P03** | **P04** | **P05** | **P06** |
| 268 | 9.918E-07 | 2.8 | P06727 | Apolipoprotein IV | 5.28 | 45371 | 2.69E+10 | 3.03E+10 | 2.95E+10 | 1.13E+10 | 1.52E+10 | 1.09E+10 |
| 248 | 2.386E-06 | 3.6 | P25311 | Zinc-alpha-2-glycoprotein | 5.71 | 34465 | 1.77E+10 | 2.29E+10 | 2.05E+10 | 7.41E+09 | 6.43E+09 | 7.20E+09 |
| 248 | 2.386E-06 | 3.6 | P00738 | Haptoglobin | 6.13 | 45861 | 1.77E+10 | 2.29E+10 | 2.05E+10 | 7.41E+09 | 6.43E+09 | 7.20E+09 |
| 264 | 6.222E-06 | 2.8 | P00738 | Haptoglobin | 6.13 | 45861 | 2.66E+10 | 3.06E+10 | 2.89E+10 | 1.10E+10 | 1.21E+10 | 1.19E+10 |
| 265 | 3.713E-05 | 3.1 | P02679 | Fibrinogen gamma chain | 5.37 | 52106 | 1.24E+10 | 1.84E+10 | 1.48E+10 | 6.04E+09 | 6.51E+09 | 6.34E+09 |
| 260 | 6.379E-05 | 2.7 | P25311 | Zinc-alpha-2-glycoprotein | 5.71 | 34465 | 3.68E+10 | 6.41E+10 | 6.49E+10 | 3.72E+10 | 2.95E+10 | 2.41E+10 |
| 260 | 6.379E-05 | 2.7 | P00738 | Haptoglobin | 6.13 | 45861 | 3.68E+10 | 6.41E+10 | 6.49E+10 | 3.72E+10 | 2.95E+10 | 2.41E+10 |
| 199 | 1.262E-04 | 8.4 | P02768 | Serum Albumin | 5.92 | 71317 | 1.62E+09 | 1.22E+09 | 7.94E+08 | 1.36E+09 | 1.10E+09 | 6.68E+09 |
| 239 | 1.269E-04 | 2.5 | P01857 | Ig gamma-1 chain C | 8.46 | 36596 | 1.07E+10 | 1.81E+10 | 1.89E+10 | 9.29E+09 | 7.49E+09 | 1.06E+10 |
| 233 | 1.302E-04 | 2.3 | P01857 | Ig gamma-1 chain C | 8.46 | 36596 | 9.74E+09 | 1.45E+10 | 1.45E+10 | 8.28E+09 | 6.42E+09 | 8.73E+09 |
| 269 | 2.343E-04 | 2.3 | P02679 | Fibrinogen gamma chain | 5.37 | 52106 | 1.29E+10 | 1.29E+10 | 1.09E+10 | 5.52E+09 | 7.34E+09 | 6.13E+09 |
| 453 | 2.446E-04 | 2.6 | P01834 | Ig kappa chain C region | 5.58 | 11773 | 1.55E+10 | 2.67E+10 | 3.96E+10 | 2.95E+10 | 1.69E+10 | 2.14E+10 |
| 399 | 2.640E-04 | 2.4 | P02760 | Protein AMBP | 5.95 | 39886 | 5.51E+09 | 5.73E+09 | 1.00E+10 | 7.27E+09 | 1.33E+10 | 9.43E+09 |
| 467 | 2.852E-04 | 2.2 | P01834 | Ig kappa chain C region | 5.58 | 11773 | 2.50E+10 | 3.71E+10 | 4.36E+10 | 3.63E+10 | 1.97E+10 | 2.82E+10 |
| 238 | 3.054E-04 | 2.2 | P01857 | Ig gamma-1 chain C | 8.46 | 36596 | 1.39E+10 | 2.21E+10 | 2.22E+10 | 1.33E+10 | 1.00E+10 | 1.31E+10 |
| 228 | 3.840E-04 | 1.8 | P35520 | Cystathionine beta-synthase | 6.20 | 61175 | 1.99E+10 | 2.40E+10 | 2.48E+10 | 1.81E+10 | 1.34E+10 | 2.01E+10 |
| 220 | 5.725E-04 | 2 | P02675 | Fibrinogen beta chain | 8.54 | 56577 | 4.74E+09 | 7.00E+09 | 6.15E+09 | 4.89E+09 | 3.59E+09 | 6.49E+09 |
| 466 | 9.355E-04 | 1.9 | P00746 | Complement factor D | 6.85 | 27529 | 3.28E+10 | 4.94E+10 | 6.24E+10 | 5.30E+10 | 3.43E+10 | 4.26E+10 |
| 266 | 0.001 | 2.6 | P02679 | Fibrinogen gamma chain | 5.37 | 52106 | 9.29E+09 | 5.91E+09 | 6.20E+09 | 4.36E+09 | 5.88E+09 | 3.52E+09 |
| 240 | 0.001 | 2.6 | P01857 | Ig gamma-1 chain C | 8.46 | 36596 | 1.12E+10 | 1.69E+10 | 1.83E+10 | 9.03E+09 | 6.97E+09 | 1.11E+10 |
| 219 | 0.001 | 1.9 | P02675 | Fibrinogen beta chain | 8.54 | 56577 | 5.25E+09 | 5.90E+09 | 5.16E+09 | 4.47E+09 | 3.30E+09 | 6.17E+09 |
| 232 | 0.001 | 1.4 | P02675 | Fibrinogen beta chain | 8.54 | 56577 | 8.14E+09 | 1.07E+10 | 1.13E+10 | 1.03E+10 | 8.05E+09 | 1.00E+10 |
| 243 | 0.002 | 2.7 | P01857 | Ig gamma-1 chain C | 8.46 | 36596 | 2.00E+10 | 2.60E+10 | 3.43E+10 | 1.66E+10 | 1.27E+10 | 2.12E+10 |
| 603 | 0.003 | 2.1 | P02765 | Alpha-2-HS-glycoprotein | 5.43 | 40098 | 3.74E+10 | 3.69E+10 | 6.74E+10 | 3.53E+10 | 3.19E+10 | 3.84E+10 |
| 231 | 0.003 | 1.6 | P02675 | Fibrinogen beta chain | 8.54 | 56577 | 6.27E+09 | 6.76E+09 | 9.80E+09 | 8.95E+09 | 8.73E+09 | 8.21E+09 |
| 230 | 0.003 | 1.9 | P01857 | Ig gamma-1 chain C | 8.46 | 36596 | 1.39E+10 | 1.84E+10 | 1.83E+10 | 1.28E+10 | 9.50E+09 | 1.44E+10 |
| 443 | 0.003 | 3 | P01834 | Ig kappa chain C region | 5.58 | 11773 | 6.30E+09 | 6.56E+09 | 1.50E+10 | 1.06E+10 | 8.35E+09 | 4.97E+09 |
| 393 | 0.004 | 2.2 | P02760 | Protein AMBP | 5.95 | 39886 | 1.04E+10 | 1.01E+10 | 1.89E+10 | 1.62E+10 | 2.21E+10 | 1.52E+10 |
| 216 | 0.004 | 2.4 | P02675 | Fibrinogen beta chain | 8.54 | 56577 | 1.15E+09 | 1.10E+09 | 5.49E+08 | 1.32E+09 | 7.76E+08 | 9.43E+08 |
| 471 | 0.005 | 1.9 | P01834 | Ig kappa chain C region | 5.58 | 11773 | 1.59E+10 | 2.15E+10 | 2.84E+10 | 2.15E+10 | 1.52E+10 | 1.56E+10 |
| 52 | 0.005 | 5.1 | P02768 | Serum Albumin | 5.92 | 71317 | 5.67E+09 | 8.00E+09 | 1.97E+09 | 1.01E+10 | 8.41E+09 | 4.10E+09 |
| 185 | 0.005 | 4.6 | P02768 | Serum Albumin | 5.92 | 71317 | 3.93E+09 | 2.95E+09 | 2.07E+09 | 2.68E+09 | 1.94E+09 | 9.00E+09 |
| 455 | 0.006 | 2.3 | P01834 | Ig kappa chain C region | 5.58 | 11773 | 1.73E+10 | 3.04E+10 | 4.06E+10 | 3.56E+10 | 2.25E+10 | 2.68E+10 |
| 227 | 0.006 | 1.5 | P02749 | Beta-2-glycoprotein | 8.34 | 39584 | 4.29E+09 | 3.96E+09 | 5.76E+09 | 6.10E+09 | 5.62E+09 | 4.17E+09 |
| 600 | 0.009 | 2.4 | P02774 | Vitamin D-binding protein | 5.40 | 54526 | 9.11E+09 | 6.88E+09 | 6.84E+09 | 5.51E+09 | 3.85E+09 | 7.80E+09 |
| 180 | 0.012 | 2.9 | O15297 | Protein phosphatase 1D | 9.14 | 67374 | 6.50E+09 | 5.28E+09 | 3.61E+09 | 4.71E+09 | 2.61E+09 | 7.69E+09 |
| 123 | 0.012 | 2.7 | P00450 | Ceruloplasmin | 5.44 | 122983 | 8.76E+09 | 2.00E+10 | 1.31E+10 | 1.10E+10 | 8.67E+09 | 7.29E+09 |
| 400 | 0.013 | 3.8 | P02760 | Protein AMBP | 5.95 | 39886 | 1.75E+09 | 1.22E+09 | 2.34E+09 | 2.61E+09 | 4.65E+09 | 1.96E+09 |
| 606 | 0.013 | 4.6 | P02647 | Apolipoprotein A-I | 5.56 | 30759 | 1.23E+10 | 1.26E+10 | 1.57E+10 | 4.33E+10 | 4.78E+10 | 1.03E+10 |
| 447 | 0.016 | 2.1 | P01834 | Ig kappa chain C region | 5.58 | 11773 | 9.85E+09 | 1.44E+10 | 2.05E+10 | 1.63E+10 | 1.05E+10 | 1.00E+10 |
| 438 | 0.025 | 2 | P01834 | Ig kappa chain C region | 5.58 | 11773 | 8.93E+09 | 9.31E+09 | 1.70E+10 | 1.81E+10 | 1.32E+10 | 9.13E+09 |
| 244 | 0.028 | 2.9 | P01857 | Ig gamma-1 chain C | 8.46 | 36596 | 1.85E+10 | 1.98E+10 | 3.16E+10 | 1.82E+10 | 1.08E+10 | 1.63E+10 |
| 263 | 0.029 | 2 | P02679 | Fibrinogen gamma chain | 5.37 | 52106 | 2.33E+09 | 1.79E+09 | 1.48E+09 | 1.19E+09 | 1.74E+09 | 1.58E+09 |
| 591 | 0.034 | 1.8 | P02787 | Serotransferrin | 6.81 | 79294 | 4.88E+10 | 4.16E+10 | 3.59E+10 | 5.98E+10 | 6.38E+10 | 5.14E+10 |
| 229 | 0.279 | 1.8 | P01009 | Alpha-1-antitrypsin | 5.37 | 46878 | 2.73E+10 | 3.85E+10 | 4.38E+10 | 4.42E+10 | 3.56E+10 | 2.41E+10 |
| 001 | - | - | P02753 | Retinol-binding protein 4 | 5.76 | 23337 | - | - | - | - | - | - |
| 002 | - | - | P02763 | Alpha-1-acid glycoprotein | 4.93 | 23725 | - | - | - | - | - | - |
| 003 | - | - | P61769 | Beta-2-microglobilin | 6.06 | 13820 | - | - | - | - | - | - |
| 004 | - | - | P02790 | Hemopexin | 6.55 | 52385 | - | - | - | - | - | - |
| 005 | - | - | P01871 | Ig mu chain C | 6.35 | 49960 | - | - | - | - | - | - |
| 006 | - | - | P02766 | Transthyretin | 5.52 | 15991 | - | - | - | - | - | - |
